# Microbiological Impact of the Use of Reclaimed Wastewater in Recreational Parks

**DOI:** 10.3390/ijerph14091009

**Published:** 2017-09-04

**Authors:** Oskar A. Palacios, Francisco J. Zavala-Díaz de la Serna, María de Lourdes Ballinas-Casarrubias, María S. Espino-Valdés, Guadalupe V. Nevárez-Moorillón

**Affiliations:** 1 Circuito Universitario S/N Campus Universitario II, Universidad Autónoma de Chihuahua, Chihuahua, Chih 31125, Mexico; oskar.palacios27@gmail.com (O.A.P.); fzavala@uach.mx (F.J.Z.-D.d.l.S.); mballinas@uach.mx (M.d.L.B.-C.); mespino@uach.mx (M.S.E.-V.); 2 Laboratorio de Microbiología Ambiental, Centro de Investigaciones Biológicas del Noroeste (CIBNOR), Calle IPN 195, La Paz, B.C.S. 20396, Mexico

**Keywords:** wastewater, recreational parks, *E. coli*, *Salmonella*, multidrug-resistant bacteria

## Abstract

Reclaimed wastewater for irrigation is an opportunity for recovery of this natural resource. In this study, microbial risk from the use of treated wastewater for irrigation of recreational parks in the city of Chihuahua, evaluating the effect of distribution distance, season, and presence of storage tanks, was analyzed. *Escherichia coli*, *Salmonella*, and multidrug-resistant bacteria were recovered from samples of reclaimed water and soils at recreational parks in Chihuahua by the membrane filtration method, using selected agars for microbial growth. Samples were taken at three different seasons. No correlation in the presence of microbial indicators and multidrug-resistant bacteria (*p* > 0.05) was found between the distance from the wastewater treatment plant to the point of use. Presence of storage tanks in parks showed a significant effect (*p* < 0.05) with a higher level of *E. coli*. The highest count in wastewater occurred in summer. We isolated 392 multidrug-resistant bacteria from water and soil; cluster analysis showed that the microorganisms at each location were of different origins. Irrigation with reclaimed wastewater did not have a negative effect on the presence of microbial indicators of the quality of soils in the parks. However, the prevalence of multidrug-resistant bacteria still represents a potential risk factor for human health.

## 1. Introduction

The increase in water demand from population increase in arid and semi-arid zones makes converted domestic wastewater a precious resource. Reclaimed wastewater can be re-used in urban areas, in agriculture, or in industrial processes. Regulations in Mexico, specifically contained in the Official Mexican Standard NOM-003-ECOL-1997, allow the uses of reclaimed water for non-potable uses, such as irrigating landscapes, public gardens, and groundwater recharge [1]. Using reclaimed water can mitigate water requirements and diminish the generation of other environmental problems [2]. Nevertheless, treated wastewater for irrigation produces increased salinity, pH, and organic content in soils of household and recreational gardens [3,4]. Wastewater treatment does not eliminate all health risks, including the spread of antibiotic-resistant bacteria that can transfer their resistance through mobile genetic elements (plasmids, transposons, and gene cassette integrons) to other microbial cells [5,6]. Transfer of resistance genes can be done by any of these mechanisms: (1) horizontal transference—incorporation into the genome of a gene from a different microorganism or from an outside source; or (2) vertical transference—which occur in the progeny by the direct transfer of genetic material from parent cells [7]. Additionally, the use of treated wastewater is not accepted as suitable for human consumption by many cultures [1].

There are integrated plans for using treated wastewater in major cities worldwide [8], which requires monitoring the quality of the reclaimed water, including microorganisms, such as *Salmonella* spp., *Escherichia coli*, and protozoa, that are used as microbial indicators of quality [8,9]. *E. coli* has been described as the most specific indicator of fecal pollution, because its natural habitat is the large intestine of warm-blooded animals, and does not survive for long periods outside of the intestinal tract [10]. Additionally, the presence of *E. coli* has been related to the potential presence of pathogens, such as *Salmonella* spp. or hepatitis A virus [11,12]. On the other hand, the presence of antibiotics and bacteria with antibiotic-resistant genes in soil and water has been designated contaminants [13] and their presence it has been identified as a serious medical problem by World Health Organization [14]. Even more, antibiotic-resistant bacteria, as indicators of contamination of the environment, was proposed as an alternative to the currently-used microbiological indicators [15]. Although many antibiotic-resistant bacteria are not pathogenic, resistant genes can be transferred to pathogens, which represents a public health risk; together with this, multidrug-resistant bacteria can persist in soils after long periods after the last irrigation with wastewater [16]. In recent years, the search for antibiotic-resistant bacteria in effluents of wastewater treatment plants have gained importance in evaluating possible environment impacts of the wastewater as a resource [17,18]. One of the main concerns is the effect of wastewater treatment biological processes on the survival of antibiotic-resistant bacteria and antibiotic-resistant gene transfer in the environment [18].

Although it has been reported that wastewater treatment systems efficiently reduce the presence of quality microbial indicators [19,20], it is possible that the distribution systems can affect the final quality of the reclaimed water, by allowing bacterial regrowth [21,22]. Moreover, the presence of storage sites, such as storage tanks at the final disposition points, tends to decrease water quality as well [23]. Accordingly, an evaluation of the wastewater effluent in the treatment plant is not enough to conclude on the safety or the possible environmental impact of using reclaimed wastewater for irrigation purposes [24]. Taking into account all the above considerations, an evaluation of the presence of microbial indicators of quality in reclaimed wastewater at the final point of reuse is recommended.

For this reason, the purpose of this study was to determine the water quality of reclaimed wastewater, as indicated by the presence of microbial indicators of quality, including *E. coli*, *Salmonella*, and multi-drug resistant bacteria in effluents received in parks at different distance from the wastewater treatment plant. Additionally, the same microbial indicators were used to indicate conditions in the soil of the parks. We tested the hypothesis that parks further from the wastewater treatment plant and those that have storage tanks for reclaimed wastewater, showed higher prevalence of quality microbial indicators and multi-drug resistant bacteria than parks closer to the wastewater treatment plant or without storage tanks.

## 2. Materials and Methods

### 2.1. Sampling

**Figure 1:**
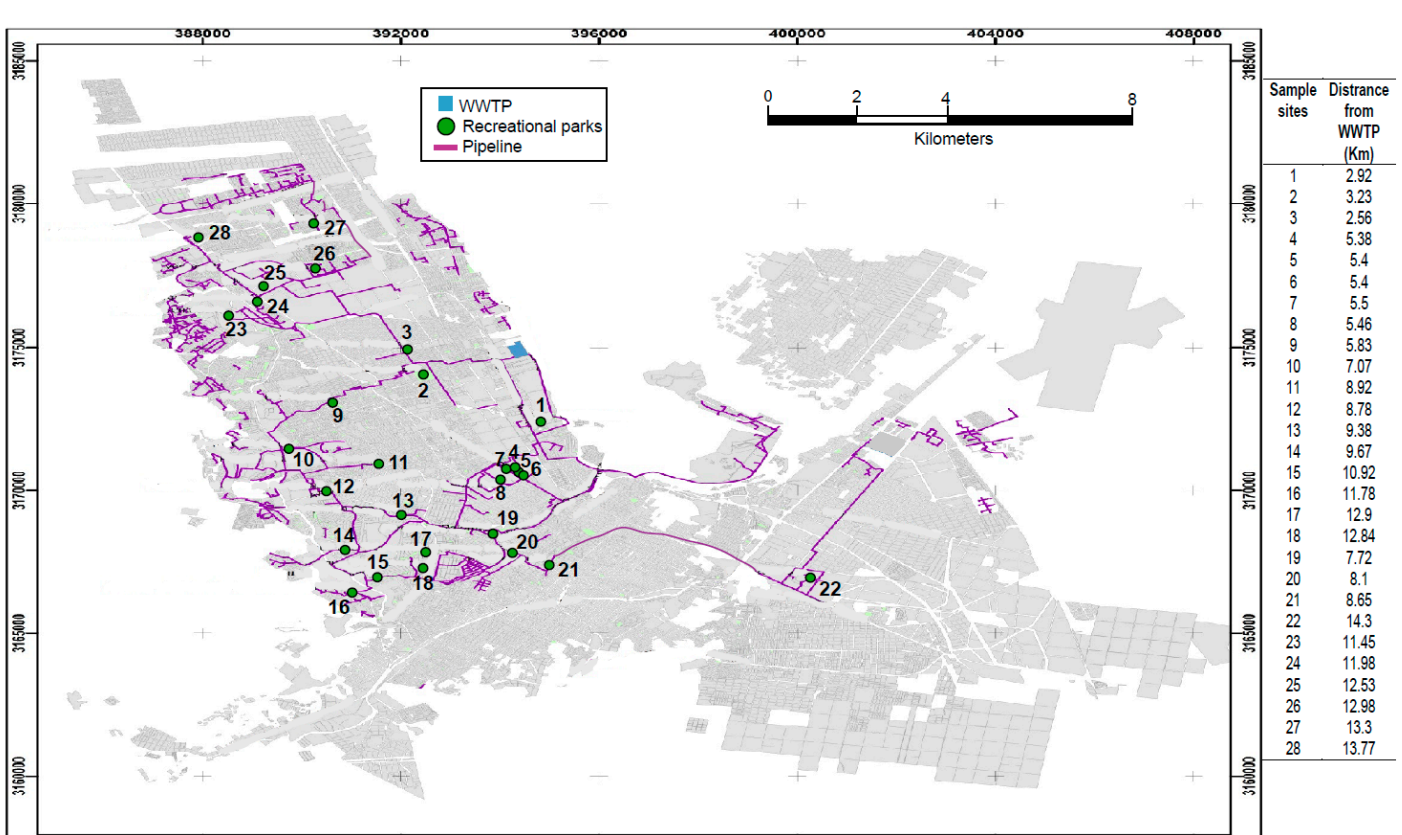
Sample sites (from Briones [26]). Recreational parks (green points) in the city of Chihuahua in Mexico irrigated by reclaimed wastewater (purple line = pipeline) from wastewater treatment plant (WWTP = blue square).

A total of 28 recreational parks in the city of Chihuahua in Mexico, which are irrigated with reclaimed wastewater from the wastewater treatment plant were sampled (Figure 1). Treatment plants in Chihuahua are level 2 facilities, where the treatment process includes mechanical pre-treatment, and an aerobic activated sludge system, followed by a disinfection process by chlorine. Samples of reclaimed wastewater (100 mL from the faucet) and the soil surface (5 g from 0 to 10 cm deep) from each park were taken, in triplicate, during three seasons in 2011: February (winter), May (summer), and September (autumn). Samples were collected in sterile containers and brought to the laboratory under refrigeration. The silty clay soils were dried in a microbiological chamber at 30 °C on an aluminum tray overnight, and then sieved through 0.15 mm mesh [25].

### 2.2. Determination of Chemical Oxygen Demand (COD)

COD was measured by oxidation with dichromate [27]. Briefly, 0.4 g of mercuric sulfate (231-992-5; Merck Millipore, Bellerica, MA, USA) and 5 mL of 0.04 M potassium dichromate solution was added to 10 mL of sample and mixed for 10 min. The remnant of potassium dichromate was -titrated with a solution 0.012 M ammoniacal ferrous sulfate using ferroin as indicator. 1 M of reduced dichromate it is equivalent to 1.5 M O_2_.

### 2.3. Initial Processing of Soil and Water Samples

Water samples were analyzed within 3 h of collection, each replicate was filtering 100 mL through a sterile cellulose membrane of cellulose (0.45 μm pore size, Merck Millipore, Bellerica, MA, USA). We used the membrane filtration method for soil analysis, which has been reported as having significant linear correlation (*r* = 0.91), compared with the MPN (most-probable number) technique to enumerate microorganisms [28]. For each replicate of soils samples, 1 g was vortexed in 100 mL of 0.85% sterile saline solution and then filtered through the 0.45 μm sterile cellulose membrane.

### 2.4. Determination of E. coli

For *E. coli* analyses, the membrane filter method was used [29]. After filtration, the membrane was placed on a Petri plate containing violet red bile agar (211695, Difco/Becton Dickinson, Franklin Lakes, NJ, USA) and then the agar was incubated for 24 h at 44.5 ± 0.2 °C. After incubation, the colonies on the membrane were counted and reported as CFU·100 mL^−1^.

### 2.5. Determination of Salmonella

For counting *Salmonella*, the membrane filter method was used [29]. After filtration, the membrane was pre-enriched in 90 mL of peptone buffer containing (in g·L^−1^): tryptone (10), NaCl (5), NaH_2_PO_4_ (9), KH_2_PO_4_ (1.5), and incubated at 36 ± 0.2 °C for 18 h. After incubation, 1 mL of peptone buffer was transferred to 10 mL of Rappaport’s medium (in g·L^−1^): soy peptone (4.5); NaCl (7.2); KH_2_PO_4_ (1.26); K_2_HPO_4_ (0.18); MgCl_2_ (13.58); and malachite green (0.036), and incubated at 41.5 ± 0.2 °C for 24 h. After incubation, a sample from Rappaport’s medium was taken and inoculated on *Salmonella-Shigella* agar and then incubated at 36 ± 0.2 °C for 24 h. The colonies suspected of belonging to *Salmonella* were confirmed by microscopy, colonial morphology, and standard biochemical tests: triple-sugar iron agar, lysine iron agar, indole ornithine motility, sulfite indole motility, methyl red, Voges-Proskauer test, and urea broth (all from Difco, Detroit, MI, USA).

### 2.6. Qualitative Determination of Multidrug-Resistant Bacteria

After filtration, the membrane was placed in a flask containing 100 mL of nutrient broth (#N7519, Sigma-Aldrich, St. Louis, MO, USA) and incubated at 36 ± 0.2 °C for 24 h. After incubation, 100 μL of inoculated nutrient broth was inoculated on Petri plates with tryptic soy agar (TSA; Difco/Becton Dickinson, Franklin Lakes, NJ, USA), which contained different antibiotics (in mL^−1^): ampicillin (10 μg); riphampicin (5 μg); chloramphenicol (30 μg); ciprofloxacin (5 μg); gentamicin (10 μg); and trimethoprim-sulphametoxazole (300 μg), and then incubated at 36 ± 0.2 °C for 24 h. Colonies were obtained in pure culture and later tested for resistance to the other five antibiotics that were analyzed. Strains were inoculated, using a sterile toothpick in a TSA plate with antibiotics, as mentioned above. Gram stain, catalase, and oxidase tests, as well as the microscopic and macroscopic morphology of each isolate, were analyzed for identification. Taking into account that a multi-drug resistant bacteria is defined as a bacterial strain that acquired non-susceptibility to at least one antimicrobial agent in three or more antimicrobial categories [30], multi-drug resistant bacteria were those that were resistant to three or more antibiotics. For strains with different morphology, the results of catalase and oxidase tests, as well as the antibiotic resistant profile, were selected for further study.

### 2.7. Statistical Analysis

All samples were taken in triplicate. The presence of *E. coli* in sites with or without storage tanks was compared by Student’s *t*-test for independent samples. The effect of distance, as well as the presence or absence of storage tanks, on the *E. coli* counts and multidrug-resistant bacteria were analyzed by Pearson’s correlation coefficient (*p* < 0.05). Comparisons between seasons were done by one-way ANOVA and then by LSD post-hoc analysis. All analyses were carried out using Minitab 17 statistical software (Minitab, State College, TX, USA). Data of multi-drug resistant bacteria from soil and water samples were analyzed by cluster analysis, using binary code (0 = sensitive; 1 = resistant) to assign data for each isolate tested, with NTsys 2.02j software (Applied Biostatics, St. Paul, MN, USA); strains that showed similar antibiotic-resistance pattern were clustered. A comparison between seasons at the frequency of antibiotic-resistant bacteria obtained for each antibiotic agent tested was compared by chi-square test.

## 3. Results and Discussion

### 3.1. Presence of Microbial Indicators of Quality in Soil and Water

**Figure 2:**
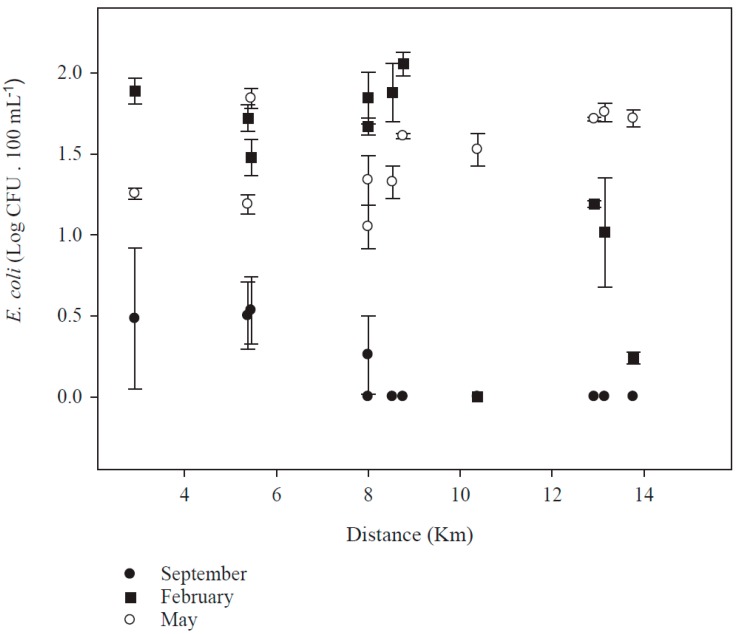
Presence of *E. coli* in reclaimed wastewater during different seasons from recreational parks at different distances from the wastewater treatment plant. Whisker lines represent the standard error.

The *E. coli* count in reclaimed water samples was different among the three seasons. The presence of *E. coli* was higher in samples taken in February and May than in September (Figure 2). Lower levels of *E. coli* in reclaimed wastewater during September could be due to the dilution effect due to the water from rains received in this month. No significant correlation between the distance of the sampling site from the treatment plant and the concentration of *E. coli* was found at any season: September (*p* = 0.88); February (*p* = 0.384); and May (*p* = 0.156). The concentration of *E. coli* in May was constant with distance and in February it decreased at >8 km from the treatment plant (Figure 2).

Distribution systems of reclaimed water in urban areas, over time, affects the final quality of treated wastewater [21,22,31]. From our tests, the distribution system that is in place in Chihuahua had no negative effect on the quality of the effluent at the point of usage. Although, there is evidence that higher temperatures enhance the bacteria count in wastewater treated with UV or chlorine [22,32], we did not find regrowth of *E. coli* in the effluent at distant points during May.

**Figure 3:**
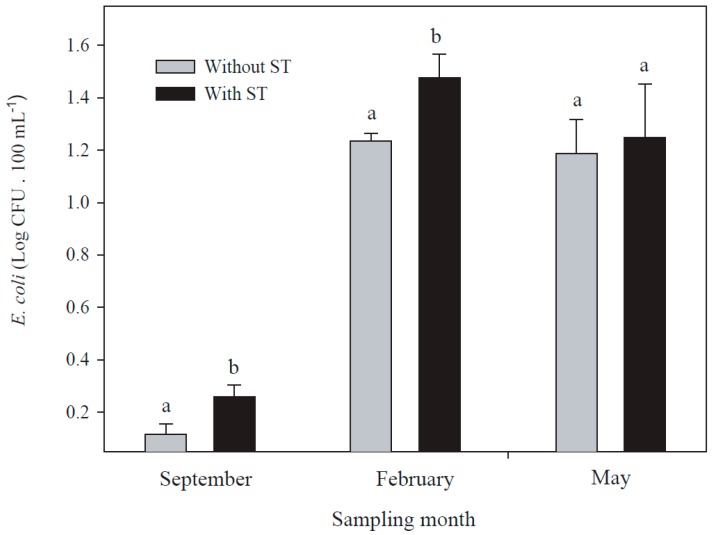
The presence of *E. coli* in reclaimed wastewater during three seasons in recreational parks with or without storage tanks (ST). Different letters indicate significant differences between conditions, using Student’s *t*-test for independent samples at *p* < 0.05. Whisker lines represent the standard error.

There is evidence that tanks storing reclaimed wastewater enhance microbial growth and biofilm and act as reservoirs for bacteria in water supplies [33]. We found a significant effect by the presence of storage tanks at the sampling point with *E. coli* during all seasons: September (*p* = 0.004); February (*p* = 0.016); and May (*p* = 0.036) (Figure 3).

**Figure 4:**
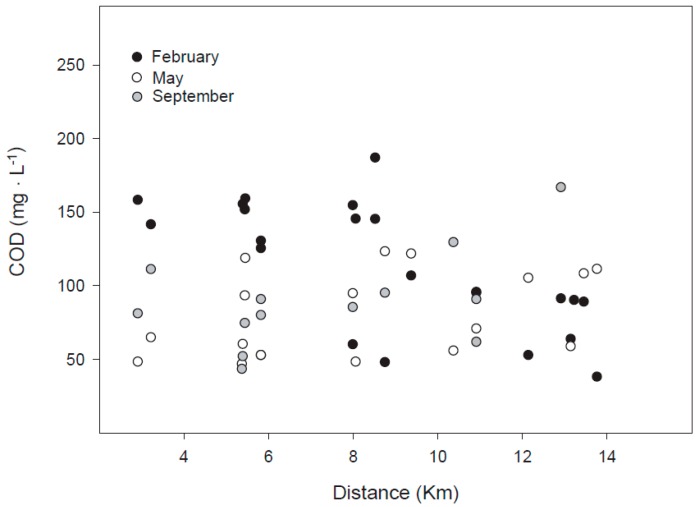
Effect of distance in the concentration of COD (chemical oxygen demand) in treated wastewater during three seasons: February (*p* = 0.009); May (*p* = 0.322); and September (*p* = 0.848).

Lye [34] found coliforms and other bacteria in water system tanks, and Al-Salaymeh et al. [23] report that poor maintenance of tanks is the main contamination. Organic matter is an important factor for the presence of microbial indicators of quality and stability of reclaimed water [35]. The amount of COD in the reclaimed wastewater decreased as the distance increases (negatively-correlated, *p* < 0.01) during February, but no correlation occurred at the other sampling dates (Figure 4). The decrease of COD with distance results from consumption of residual organic matter by microorganisms in the distribution system. In all months of sampling, the amount of COD were independent of distance between the treatment plant and the parks (data not shown). These results agree with Manios et al. [36] who reported that the distance traveled by water though the pipeline from the treatment plant and the final destination does not affect COD concentration.

**Figure 5:**
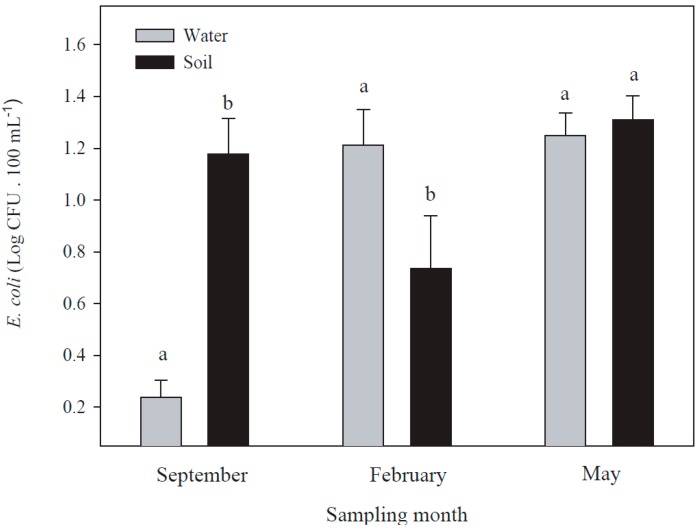
The presence of *E. coli* in reclaimed wastewater and soils in recreational parks during three seasons. Different letters indicate significant differences between conditions, using Student’s *t*-test for independent samples at *p* < 0.05. Whisker lines represent the standard error.

The effect of irrigation with reclaimed wastewater on fecal count has been controversial, but depends on soil characteristics [37]; organic matter content, pH, and moisture-holding capacity determine the retention of microorganisms in soils [38]. The effect of rain and dry weather on microbial indicators has been well established [39,40]. We did not find a relationship between the amount of *E. coli* in the reclaimed wastewater and the enumeration of this microorganism in the soils that were irrigated with this water during the months of February and September (Figure 5), however, there was a positive relation between *E. coli* counts in water and soils in summer (Figure 5). This suggests that reclaimed wastewater treated by chlorination on microbial counts in soils is more affected by environmental conditions than by the concentration of bacteria in the reclaimed water used for irrigation.

Wastewater is one of the main sources of transmission of *Salmonella* to the environment [41]. To decrease the persistence of *Salmonella*, Ravva and Sarreal [42] proposed holding the wastewater for sufficient reduction cycles in ponds. We found *Salmonella* only in one water sample (sample site 23, Figure 1) and three soil samples in sufficient amounts for qualitative determination (sample sites 3, 5, and 19, Figure 1). Although *Salmonella* can grow in grass-covered soils [43], its persistence in soils irrigated with wastewater is no more than three days [44] because it is affected by temperature, moisture, soil type, presence of plants, exposure to sunlight, and predation by protozoans and indigenous soil microbes [45]. Additionally, the source of bacteria, like *Salmonella,* may be domestic or wild animals. Still, only four samples indicated the presence of *Salmonella*.

### 3.2. Multidrug-Resistant Bacteria

Urban wastewater at treatment plants does not reduce or change the antibiotic-resistance of bacteria in the final effluent, but can reactivate and select antibiotic-resistant bacteria [31,46,47]. We isolated 392 multi-drug resistant strains in culture media at higher concentrations of each antibiotic than the Enterobacteriaceae breakpoints given by the European Committee on Antimicrobial Susceptibility Testing (EUCAST). Although the sampling sites (sampling points 4–6) that showed in soils a higher number (24, 24, and 27 isolates, respectively) of multi-drug resistant bacteria are near to WWTP (>6 km) no correlation was found between the number of multidrug-resistant bacteria in the reclaimed wastewater of these sampling points (4, 9, and 9 isolates, respectively) and the distance between the treatment plant and the sampling station (*p* = 0.761), or the presence or absence of storage tanks at sampling sites (*p* = 0.53). Many bacterial isolates from water were resistant to ampicillin and gentamicin (230 and 229 isolates, respectively) and soils (166 and 171 isolates, respectively) (Table 1). Huang et al. [31] report that chlorination during wastewater treatment reactivates antibiotic-resistant bacteria, specifically ampicillin. Reactivation could explain the prevalence of ampicillin-resistant bacterial strains in water. Birošová et al. [17] report that most coliforms in treated wastewater have a higher resistance to ampicillin and gentamicin. Additionally, intrinsic ampicillin resistance by the presence of the AmpC β-lactamase has been found in Enterobacteriaceae [48]. During September a lower presence of antibiotic-resistant bacteria was observed in water samples. Nevertheless, soils samples showed a decrement in the presence of this type of bacteria during the month of May, but not in September (*p* < 0.05) (Table 1). The effect of seasons in the prevalence of antibiotic resistant *E. coli* has been reported by Akiyama and Savin [49] in reclaimed wastewater with a significant decrement during September. On the other hand, it has been reported that in winter the prevalence of antibiotics in soils is higher than in summer [50]. Nevertheless, independently of the gain or intrinsic resistance to a specific antibiotic in bacteria, the number of bacteria with a broad antibiotics resistance profile indicates an impact from humans or animals [51,52].

Using reclaimed wastewater to irrigate soils may increase the risk of antibiotic-resistant soil microorganisms. The generation of new antibiotic-resistant microorganisms in environments containing wastewater is related to two mechanisms: (1) the presence of low levels of antibiotics in the effluents [53], and (2) the transfer of antibiotic-resistant bacteria from wastewater to the environment [18,54], which transfer their resistance to other microorganisms [55]. The real effect of wastewater irrigation on levels of antibiotic-resistant bacteria in soils is not clear. Negreanu et al. [56] indicate that irrigation with treated wastewater of soils in crop production has a negligible effect on the levels of antibiotic-resistant bacteria in the soil.

To analyze the resistance patterns in the bacterial isolates and test the correlation between water and soil samples taken at the same sampling point, a cluster analysis was performed. Our results showed that, although both sources (water and soil) of multidrug-resistant strains presented higher resistance to the same antibiotics (ampicillin and gentamicin), we found that 249 strains of the 392 analyzed had differential resistance to the different combinations of antibiotic tested. Multidrug-resistant strains clustered by types of sample (water or soil) instead of by sampling point (Table 2).

These data indicate that the microorganisms had a behavior of antibiotic resistance that is related to the niche from which they originate (reclaimed wastewater or soil). Although there are many environmental factors that can facilitate the emergence of antibiotic resistance in bacteria, the presence of resistant genes associated with mobile genetic elements (mobilomes) make the study of the spread of antibiotic-resistant bacteria more complicated [57]. In practice, gene flow is probably structured by ecology, with species that share similar niches drawing antibiotic resistance from similar gene pools [57,58]. High levels of multidrug-resistant bacteria in soils using reclaimed wastewater represent a public health risk; the pool of antibiotic resistance genes will continue to increase [59].

## 4. Conclusions

The use of reclaimed wastewater for irrigating city parks does not present an immediate effect on the presence of microbial indicators of quality in soils, as determined by indicator microorganisms, such as *E. coli* and *Salmonella*. Our results show the relationship between season, distance from the treatment plant, and storage tanks for reclaimed wastewater at the sampling point with *E. coli* and multidrug-resistant bacteria. The hypothesis tested in this work is rejected for the effect of distance on microbial indicators, but accepted for the presence of storage tanks in the parks. The effects of summer on the concentration of *E. coli* demonstrate the importance of controlling chlorine concentration at the treatment plant to avoid possible increases in levels of bacterial growth. Although cluster analysis indicates that multidrug-resistant bacteria are clustered according to their origin in water or soil, the high levels of these microorganisms is a potential risk factor for human health.

## Figures and Tables

**Table 1:** Number of bacterial isolates resistant to each antibiotic from samples of water and soil taken at recreational parks during three seasons.

Antibiotic (μg·mL^−1^)	Water				Soil			
	September ^1^	February	May	Total	September	February	May ^1^	Total
Ampicillin (10)	64	86	80	230	65	72	29	166
Gentamicin (10)	63	84	82	229	68	74	29	171
Trimethoprim-Sulphametoxazole (300)	49	74	73	196	51	53	23	127
Chloramphenicol (30)	31	66	62	159	52	53	21	126
Riphampicin (5)	55	84	80	219	62	73	25	160
Ciprofloxacin (5)	29	62	64	155	23	29	12	64

^1^ Statistically different (*p* < 0.05), analyzed by chi-square test.

**Table 2:** Cluster of multidrug-resistant bacteria isolated from water and soils, based on the kind of antibiotic presenting resistance.

Cluster	Number of Strains	Source	
		Water	Soil
I	3	1	2
II	5	0	5
III	8	0	8
IV	8	2	6
V	28	0	28
VI	49	45	4
VII	67	66	1
VIII	81	12	69
Total	249	126	123
